# An evaluation of completed and averted school shootings

**DOI:** 10.3389/fpubh.2023.1305286

**Published:** 2024-01-09

**Authors:** Ashley T. Winch, Kristi Alexander, Clint Bowers, Frank Straub, Deborah C. Beidel

**Affiliations:** ^1^Department of Psychology, UCF RESTORES, University of Central Florida, Orlando, FL, United States; ^2^Department of Psychology, University of Central Florida, Orlando, FL, United States; ^3^Safe and Sound Schools, Newtown, CT, United States

**Keywords:** school shooting, prevention, mass violence, education, averted

## Abstract

**Introduction:**

For over two decades school shootings have become a significant concern, especially in the United States. Following a rampage school shooting, extensive resources are devoted to gathering all of the information surrounding the event. To date, few studies have compared completed to averted, or near-miss, school shootings. This study utilized the largest known sample of cases based in the United States in an effort to identify potential targets for prevention.

**Method:**

Data were derived from the *Averted School Violence* database of incidents occurring between 1999 and 2020. Statistical analyses were conducted to determine how age, co-conspirator involvement, engagement in leakage warning behavior, and motives – in isolation and in combination – varied between groups.

**Results:**

In insolation, age, co-conspirator involvement, engagement in leakage warning behaviors, and motives were significantly different between groups. However, when these variables were combined into a logistic regression, co-conspirator involvement, engagement in leakage warning behaviors, and motives involving suicidal intent emerged as statistically significant predictors of group membership. Age no longer differentiated the two types of events.

**Conclusion:**

This study demonstrates that regardless of suspect age, threats of school violence must be taken seriously and investigated fully. Further, students reporting their peers’ engagement in shooting-related behaviors (e.g., bringing a gun to school, mapping school, etc.) was one of the most significant predictors that a plot will be thwarted. While perpetrators who planned with others had increased odds of their plot being identified, those acting alone still demonstrated leakage behaviors. If individuals in the school environment are educated regarding warning behaviors, lone perpetrators can still be identified and reported to authorities. The perpetrator’s emotional distress, in particular depressive or suicidal thoughts were also a significant predictor of a completed school shooting. Future research efforts should focus on the development and evaluation of peer training programs to assist in the detection of school shooting warning behaviors.

## Introduction

One of the threats to safety in schools is gun violence. In 1999, “Columbine” became synonymous with “school shooting.” Over the last two decades, the number of school shootings has increased, with Virginia Tech, Sandy Hook, Parkland, and Uvalde, among the deadliest. The current rates of school shootings are the highest in history (1). Although mass shootings occur in various public places, schools are one of the most common locations for gun violence (2–4). Understanding the factors contributing to this type of school violence is a critical first step to address this public health crisis.

For the last two decades, numerous studies have examined characteristics of completed school shootings, including the perpetrator’s mental health status, history of bullying or disciplinary issues, family status, and the level of social connection to the school (5–10). Despite these investigations, one finding remains consistent: perpetrator characteristics fail to predict who will be the next school shooter (10–13). One explanation may be that examining this problem solely through the lens of completed school shootings may yield a generic perpetrator profile. Many individuals share the characteristics attributed to school shooters (e.g., male, having been bullied at school, facing adverse experiences in childhood, etc.), yet they never contemplate or complete a school shooting. Therefore, although continuing to define perpetrator characteristics is important, understanding the contextual factors that surround school shootings and the incidents that are thwarted can provide a more complete picture.

Although there is debate about how to define school shootings (14), the definition adopted by the Averted School Violence (ASV) Project is among the most common and consistently used in other investigations (3, 15, 16). An averted school shooting is defined as “a planned school attack, with or without the use of a firearm, that was prevented either before or after the perpetrator’s arrival on school grounds, and before any injury or loss of life occurred.” Cases are classified as completed if there was at least one injury [(17), p. 7].

Despite the consistency in classification, there are few studies comparing averted and completed school shootings. A 2019 preliminary report released by the National Policing Institute (NPI; formerly the National Police Foundation) that utilized the ASV database, found that public schools, high schools, and schools located in suburban areas were targeted most often, regardless of completion status (18). Additionally, suspects in both completed and averted shootings were most often current or recent students at the targeted school (18). Motivations behind the shooting plots were also consistent regardless of completion status. These motives included hatred toward others, grudges or revenge seeking, bullying, envy, and resentment. However, completed shootings also had additional motives, including paranoid delusions or command hallucinations [a type of auditory verbal hallucinations characterized by voice commanding content, instructing the person to follow harmful commands; (18, 19)]. With respect to other differences, the NPI found a stratification between the groups when considering suspects’ age and number of accomplices. Specifically, averted cases most often involved suspects between the ages of 14 and 15, while in completed cases, suspects were most often in their 20s or 40s. In this study (18), approximately 41 percent of averted cases involved co-conspirators, which is a departure from the most recently published (20) study, where. Approximately 90 percent of the averted cases involved a person acting alone. This difference between the 2019 and 2021 studies may be accounted for by the increased number of cases in the sample (*N* = 51 versus *N* = 170, respectively).

Despite being unable to predict who will complete a school shooting (11), what is clear is that perpetrators, for the most part, are not entirely inconspicuous. They often display warning behaviors before carrying out their plan (11, 16, 21, 22). Warning behaviors fall into eight categories: *Leakage* (told someone)*, Pathway* (planning and preparation)*, Fixation* (preoccupation with a person or a cause)*, Identification* (taking on a “warrior mentality”)*, Novel Aggression* (novel act of violence unrelated to the target)*, Energy Burst* (increased frequency/variety of behaviors toward the target)*, Last Resort* (violence out of desperation)*, and Directly Communicated Threat* (telling targets of their intention to harm them; for more information, see (23)). In an investigation of shootings in German schools that compared completed school shootings (*N* = 9) to shootings that were averted prior to the acquisition of weapons (*N* = 31), leakage was demonstrated by all the perpetrators. All perpetrators that carried out school shootings additionally engaged in pathway, fixation, and identification warning behaviors, and most demonstrated last resort warning behavior (24). While each warning behavior is of interest, this study focuses on leakage warning behavior (see Method section for rationale).

One frequently asked question following a school shooting is motive (why?). In both completed and averted cases, grievance was the primary motivation demonstrated by perpetrators (11, 18, 22). A desire to kill was the second most prevalent motive identified in completed (11) and averted school shootings (22). While less common, suicidal ideation, fame seeking, and psychotic symptoms were motives that were found across samples (11, 18, 22). In averted cases, anti-female sentiment and white supremacy were additional motives behind the plots (22). Understanding these motives and being able to identify related behaviors displayed in advance may be an important factor in averting school shootings.

In summary, school violence is a serious public health problem, but to date, there is limited information about the risk factors that may help prevent school shootings or attempts. The purpose of the current study was to expand on the previous literature by determining whether variables other than shooter demographics differentiated averted versus completed school shootings using an available, open-source database. Given the natural constraints that come from utilizing open-source data and laws around reporting information when the suspect is a minor, only variables consistently available across numerous public reports were included in the analysis. These variables included the age of the perpetrators, number of perpetrators, engagement in leakage warning behaviors, and the motives behind the shooting plots. It was hypothesized that, on average, suspects in averted school shootings would be (1) younger, (2) have a co-conspirator, (3) engage in leakage warning behavior, and (4) have a grievance related motive. To our knowledge, this is the first investigation to examine motives and leakage warning behavior as potential discriminators of averted versus completed school shootings.

## Methods and materials

### Database

The ASV database represents a collaboration between school safety subject matter experts and numerous national and state-level organizations. It consists of incident-level information about averted or completed attacks, lessons learned, and potential safety strategies that may be implemented to prevent future attacks. The database includes both open-source information collected by staff at the National Police Foundation and accounts shared by those directly involved in the averted or completed attacks, whether it be school personnel or law enforcement officers. The ASV database is now administered by Safe and Sound Schools, a non-profit school safety organization founded after the Sandy Hook Elementary shooting.^1^ In this investigation, we examined whether any of the following variables: age, presence of accomplices, engagement in leakage warning behaviors, and motives could discriminate between an averted and a completed school shooting. Each variable was evaluated separately and then combined into a logistic model.

### Power calculation

One-hundred forty-nine averted school shooting cases and 80 completed school shooting cases were included in this analysis. Due to the limited research investigating completed and averted school shootings, there are a lack of data to inform sample size estimations. As a result, the suggestions outlined in Peduzzi et al. (25) were used. Four independent variables were evaluated using the “rule of thumb” (for each variable, they suggest a minimum of 10 observations). Second, G*power version 3.1 was used to determine the sample size, as indicated by Faul et al. (26). Power was calculated based on a medium effect size of 0.15. Based on the guidelines outlined in Cohen (27) and Cohen et al. (28) a minimum sample size of 85 cases was deemed appropriate for the planned statical analyses.

### Measures and procedures

There were 262 cases available in the ASV database. To collect the data, the National Police Foundation staff analyzed news articles related to school-based attacks that were both completed and averted, using Google internet searches for recent averted or completed school based attacks. When possible, additional information was provided by people involved in the incident. All information was deidentified to protect the privacy of those involved. Experts also evaluated the cases to identify factors such as pathway warning behavior. The primary alleged shooter’s information was used in cases with more than one shooter.

Cases were included in the current study if the shootings or the shooting plots were located on school grounds (primary schools, secondary schools, and colleges), involved the use or planned use of a gun, and were not related to organized crime violence (e.g., gang or community-related violence carried onto school grounds). Thirty-three (33) cases were eliminated because they did not meet the criteria for inclusion. Attacks were labeled as completed if they occurred on school grounds during school hours or at a school-related function after school hours and resulted in at least one injury. An averted attack was included if there was evidence that a person engaged in any pathway (i.e., researching previous school shootings, buying a gun, writing down a plan for an attack) behavior related to preparation for a school-based attack. The sample was predominantly male (90.0 percent), with ages that ranged from 12 to 62 (*M* = 20.09, *SD* = 9.20). Most suspects were current (68.6 percent) or former students (18.8 percent) at the target school.

As noted above, variables included in the analysis were age (coded in years), number of perpetrators, presence/absence of warning behaviors, and motive. Although it had been the original intent to examine all eight types of warning behaviors, leakage was the only category documented consistently in the ASV database. Unsurprisingly, leakage is the most commonly demonstrated warning behavior (23, 24, 29) in both completed (11, 23), and averted school shootings (16, 20). Specifically, “Leakage Warning Behavior” is defined as any instance when a perpetrator discloses their plan to carry out a school shooting to persons who are not the intended target(s).

With respect to motive, each case was evaluated and coded based on one of nine predetermined categories. The categories were developed by three field experts and may be found in Table 1. The motive definitions were similar to those outlined in the 2019 U.S. Secret Service Analysis of Targeted School Violence (11). The final motive definitions and decision rules outlined in Appendix A were created as a collaborative effort of researchers at UCF RESTORES at the University of Central Florida and Safe and Sound Schools. After removing cases that did not meet inclusion criteria, 179 cases contained sufficient information to be coded by a single field expert. The field expert was the first author, a fifth-year clinical psychology doctoral student with a background in trauma, and was trained to evaluate both averted and completed cases by the 4th and 5th authors. The field expert had three years of experience in case evaluation prior to completing this task. In the instances where the motive was not apparent to the expert, the case was moved to a separate file and coded by two other field experts, who were doctoral level psychologists with a background in trauma. The additional field experts coded a total of 50 cases for a total of 229 cases available for motive analysis. When there was a discrepancy in classifying the motives, the original field expert served as the “tiebreaker” and would select the motive based on the subset identified by all the field experts.

**Table 1:** Motive categories.

Motive	Definition
Grievances	Any form of grievance, whether it be towards a member of school personnel, students, or just people in general. Cases in which a person had a specific “hit list” are included here. Additionally, in this category, there are cases that use phrases “hating everyone at the school” in reference to people not based on race or religious affiliation.
Suicide	Any case where suicide ideation, death by suicide, or suicide by cop was present, and the suspect also intended to or carried out the killing/injuring of others. This score will be prioritized over other classifications.
Mass murder	Any case in which the suspect’s goal was to kill a lot of people, but does not indicate it is out of retaliation, and no suicidal ideation is present. Cases can include those that mention performing acts like “Columbine,” “Sandy Hook” or one of the other more famous school shooting cases.
Fame	Any case in which the suspect’s goal was to kill people to become infamous/famous.
Extremist ideation	Desire to commit an attack against a specific group of people (e.g., based on race, sexuality, gender, or religion) due to extremist affiliation or ideation (e.g., White Supremacists, ISIS, Nazi, Incels, etc.).
Delusional ideation or hallucinations	Any case in which delusions or hallucinations are present. Cases included are those in which the perpetrator reports hearing “voices” belief that they are doing the work of a “higher power” or were a “higher power”
Other	Any cases that contain enough information but do not fit into one of the above categories.
Unknown	Give this code for cases in which there is not enough information to accurately categorize the case.
Do not include	Does not meet the criteria of an averted or completed school shooting and should be excluded from the database.

## Results

Analyses were conducted using SPSS Version 27. Prior to analyses, data were examined for potential issues with skewedness, multicollinearity, and other violations of statistical assumptions. The data met all necessary assumptions, and no alterations were necessary. The final sample included 229 cases, with 149 averted cases and 80 completed cases. Of the cases, 9.2 percent were missing suspect age, 3.1 percent were missing information on if a co-conspirator was involved, no cases were missing leakage warning behaviors, and no cases were missing data regarding leakage warning behavior. Given the limited missing data, all analyses utilized the simple listwise deletion method.

### Age of perpetrators

Perpetrator age was available for 129 primary suspects in the averted school shooting group and 79 in the completed school shooting group. The results of a one-tailed *t*-test revealed that the average age of the suspects in the averted group (*M* = 18.86, *SD* = 7.78) was significantly younger than the average age of the completed group (*M* = 22.09, *SD* = 10.90; *t* (206) = −2.30, *p* < 0.05; [95 percent CI −6.00 to −0.50]; *d* = 0.36).

### Accomplices

A total of 149 averted cases and 80 completed cases contained data on the presence/absence of accomplices. A chi-square test revealed that a significantly higher percentage of the averted cases involved at least one accomplice (19.0 percent) compared to completed cases [3.8 percent; χ^2^(1) = 10.20, *p* < 0.001].

### Participation in leakage behavior

Leakage warning behavior was available for 149 averted cases and 80 completed cases and was analyzed as a dichotomous variable (yes/no). There was a significantly higher percentage of warning behaviors in the averted group (82.4 percent) vs. the completed cases [17.6 percent; χ^2^(1) = 27.04, *p* < 0.001].

### Perpetrator motive

A chi-square analysis was conducted to determine whether group differences existed in perpetrator motives for planning/completing an attack. Thirty-four (34) cases were coded as having unknown motives and were excluded from this analysis. An additional 7 cases were coded as “other,” and 2 were coded as being motivated by extremist ideation. Because these motives were found so infrequently, these cases were also excluded from the analysis, leaving 186 cases. In the comparison of motives for completed vs. averted school shootings, The overall model was statistically significant χ^2^(3) = 25.22, *p* < 0.001. Out of the four motive categories included in the analysis, only Grievance, Suicide, and Mass Murder had significantly different distributions between the averted and completed cases compared to expected counts. Grievance was determined to be the motive in 27.4 percent of averted cases and 10.2 percent of completed cases. Suicide was the motive in 14.0 percent of averted cases and 21.0 percent of completed cases. Mass Murder was the motive for 17.7 percent of averted cases and 3.8 percent of completed cases. The distribution of the Delusional Ideation motive did not differ significantly across the averted and completed groups from its expected count. More information regarding the percentage of each motive across the averted and completed group may be found in Table 2.

**Table 2:** Motive percentages.

Motive	Averted	*N*	Completed	*N*
Grievance	27.4	51	10.2	19
Suicide	14.0	26	21.0	39
Mass murder	17.7	33	3.8	7
Delusional ideation	2.7	5	3.2	6

Chi^2^ Model *p* < 0.001.

### Prediction of group membership

Age of the perpetrators, presence of accomplices, participation in leakage warning behaviors, and perpetrator motive were entered into a logistic regression to examine ability to predict group membership. The Box and Tidwell (30) procedure confirmed that age was linearly related to group classification, and no further corrections needed to be made.

The overall model was statistically significant, χ^2^(6) = 55.15, *p* < 0.001, explaining 38.2 per^c^ent (Nagelkerke R^2^) of the variance in school shooting cases. Of the 165 cases available for this evaluation, 70.3 percent were correctly classified. For the individual groups, 83.2 percent of the averted cases and 52.9 percent of the completed cases were correctly identified. Among the potential predictors, accomplices, perpetrator motives, and leakage warning behaviors were statistically significant. Leakage warning behaviors, *b* = −1.82, Wald χ^2^(1) = 20.69, *OR* = 0.16, *p* < 0.001, and having a co-conspirator, *b* = −1.60, Wald χ^2^(1) = 4.90, *OR* = 0.20, *p* < 0.05, were significantly more likely to occur in the averted group in comparison to the completed group. A perpetrator with a suicide-related motive was significantly more likely to be in the completed versus averted cases, *b* = 1.53, Wald χ^2^(1) = 11.74, *OR* = 4.61, *p* < 0.001. For a complete overview of the logistic regression results, see Table 3.

**Table 3:** Results of a logistic regression analysis examining the predictive ability of the variables age, co-conspirators, and motives in classification of averted and completed shooting cases.

Variable	B	S.E.	Wald	*p*	OR	95% CI
Suspect age	0.01	0.02	0.22	0.64	1.01	0.97–1.05
Co-conspirator	−1.60	0.73	4.90	0.03*	0.20	0.05–0.83
Leakage	−1.82	0.40	20.69	<0.001 ***	0.16	0.07–0.36
Suicide–Grievance	1.53	0.45	11.74	<0.001 ***	4.61	1.92–11.04
Mass murder–Grievance	−0.22	0.57	0.15	0.70	0.80	0.26–2.43
Delusional ideation–Grievance	0.54	0.75	0.52	0.47	1.72	0.39–7.48
Constant	−0.15	0.51	0.08	0.78	0.87	

*p < 0.05, **p < 0.01,***p < 0.001.

In a small number of cases, school shootings were acts of workplace violence (e.g., disgruntled employees or a problematic romantic relationship). In order to more closely examine factors related to school-age perpetrators, we removed perpetrators above the age of 19 from the sample and conducted a new logistic regression. The overall model with adult perpetrators removed was also statistically significant χ^2^(5) = 35.56, *p* < 0.001, explaining 36.90 percent (Nagelkerke R^2^) of the variance. Of the 114 cases evaluated in the reduced sample; 76.50 percent were classified correctly. Specifically, group membership was determined accurately in 83.80 percent of the averted cases and 62.50 percent of the completed cases. Within this reduced sample, having a co-conspirator was no longer a significant predictor of group membership, however, leakage warning behavior was a significant predictor, *b* = −2.00, Wald χ^2^(1) = 17.27, *OR* = 0.14, *p* < 0.001. Once again, leakage warning behavior was more likely to be found in averted cases compared to completed cases. However, the motive variable was no longer statistically significant, therefore the individual motives could not be evaluated for statistical significance. For full results of the logistic regression, see Table 4.

**Table 4:** Results of the logistic regression analysis carried out in Table 2 restricted to those 19 years of age or younger.

Variable	B	S.E.	Wald	*p*	OR	95% CI OR
Suspect age	−0.16	0.13	1.46	0.23	0.85	0.66–1.11
Co-conspirator	−1.42	0.73	3.80	0.05	0.24	0.06–1.01
Leakage	−2.01	0.48	17.27	<0.001***	0.14	0.05–0.35
Motives			5.89	0.05		
Suicide–Grievance	1.26	0.53	5.64	0.02*	3.51	1.25–9.89
Mass murder–Grievance	0.34	0.67	0.25	0.62	1.40	0.38–5.19
Constant	2.60	2.09	1.52	0.21	13.46	

*p < 0.05, **p < 0.01, ***p < 0.001.

## Discussion

### Summary and key findings

Despite the media attention over the last two decades, much of the research on completed school shootings has focused almost exclusively on the demographic and social characteristics of the perpetrators. To our knowledge this study has the largest number of averted and completed school cases and is the first to use logistic regression to identify group differences in an attempt to inform more robust prevention efforts. The results of this investigation revealed some potential discriminators that may inform future research and prevention efforts.

Consistent with other reports in the literature [(15, 18, 31); Daniels, 2019] the results of this investigation indicated that when compared to perpetrators who completed a school shooting, perpetrators of averted school shootings were significantly younger in age. These findings were consistent whether the sample was limited to the United States [(18); Langman and Straub, 2019] or consisted of data from other nations (15). There may be several reasons why averted school shootings are associated with a younger age. First, the capacity of younger children to purchase or otherwise acquire a gun may be more restricted. The type of attack also plays a role. Targeted attacks, where the shooter has a particular person or group in mind are typically committed by individuals older than age 30 (18). Many completed school shootings are not targeted (10, 12) but are still planned in advance. Althari et al. (11) list 10 behaviors that could go into planning the attack, including weapons research and selection, deceptive practices, surveillance of or researching the target, planning the execution, and researching prior attacks [see (11) for the complete list]. These behaviors require executive functioning ability, which may not yet be fully developed in younger children. However, executive functioning skills increase with age and the ability to plan follows a developmental course with improvement throughout late childhood and into adolescence (32). Thus, younger children’s inability to plan effectively may be a second factor in why averted school shooters are younger in age.

However, age was not a statistically significant predictor of group membership when combined with other variables in the logistic regression, meaning that age alone, or in combination with the factors examined in this study, does not help prediction of who will complete a school shooting. Some in the community discount younger ages as having the ability to procure a gun. Others cite attempts to increase the age for legal purchases as one way to stop school shootings. Certainly, any means that would restrict access to lethal weapons would help. However, recent events remind us that even a 6-year-old can take a gun to school and shoot a teacher (33). Therefore, even though age restrictions for gun purchase may make it more difficult for younger children to obtain access to firearms, youth under the legal age requirement can and do acquire weapons (34). These findings indicate that, regardless of age, all threats of school violence must be taken seriously.

The involvement of more than one person in a school shooting plot was a statistically significant predictor of group membership when evaluated alone and in the regression model of the full sample. When the sample size was reduced to only include school-age perpetrators, having a co-conspirator was marginally significant (*p* = 0.05). Again, the results of this investigation are consistent with the extant literature (15, 18). Whereas the results of this investigation, as well as that of Langman and Straub (18) were based on school shootings in the United States, Agnich (15) had a slightly different database – including multiple victim attacks that resulted in at least one homicide. The latter sample was also international in scope, including incidents that occurred in 38 nations. Yet, despite the different sample characteristics and different definitions of mass violence, the results were similar. In short, across different samples and definitions, there appears to be substantial and consistent evidence that averted cases are more likely to involve engagement with co-conspirators. This involvement of co-conspirators may lead to more opportunities for others to discover the plan, and in some cases, it is the co-conspirator(s) that will report the plot. As will be discussed below, knowledge of a plot is only the first step toward averting these events. Knowing how and to whom to report is also necessary and requires further consideration.

In the examination of motives, *grievance, mass murder,* and *suicide* motives had significantly different distributions across averted and completed cases. However, having suicidal intent was the only motive that achieved statistical significance in the logistic regression, with the ability to predict the potential completion of a school shooting. These data are consistent with other investigations (11, 18, 20, 22). For example, Alathari et al. (11), using a smaller database of 41 targeted school attacks, reported that grievances against someone at the school was the most frequent primary motive, followed by desire to kill. Suicide was the third most common primary motive. However, this study also found that suicide was the most common secondary motive followed by grievance, and desire to kill. This report concluded that most attackers had multiple motivations for the targeted violence, a conclusion also reached by other reports (18, 20, 22). In contrast, in this study the focus was on factors that differentiated completed versus averted school shootings, we were interested in only the primary motive.

An important distinction of this study was the comparison of motivations between averted and completed shootings. One interpretation of these data are that grievance was the most frequent motive among suspects in averted school shootings, who may view a shooting as a means to solve a transient problem. These grievances are likely to be interpersonal in nature, and more known to others, therefore more likely to be reported to authorities. In contrast, perpetrators of completed school shootings may believe that carrying out a school shooting is a solution to their own intrapersonal problems. The finality of their actions suggests that they may have suffered for an extended period of time prior to the shooting. Such reasoning is consistent with previous investigations of completed school shootings that found evidence of depression and suicidal ideation in perpetrators’ histories (7, 9, 10, 35, 36).

Extending findings from other research, this investigation found that suspects in averted cases engaged in leakage warning behavior significantly more frequently than perpetrators of completed cases. While this may seem intuitive, given that these instances were thwarted, it is important to remember that leakage warning behavior was evident in the majority of completed cases, which constituted the entire, and much smaller, databases of prior research (10, 11). Therefore, it may be that perpetrators of completed shootings also demonstrated leakage but that no one reported this information to the appropriate authorities. Given that leakage warning behavior is one of the variables that consistently differentiated the two groups, substantial efforts should be placed into understanding how and where leakage warning behavior presents, and how to educate peers in reporting observed or reported leakage behaviors.

Although age, number of accomplices, leakage warning behavior, and motives demonstrated significant differences in individual comparisons, when evaluated in combination, the results were different and yet, very informative. While it was hypothesized that shootings were more likely to be averted because the potential perpetrators were younger, worked with accomplices, disclosed information regarding their plans, and attempted to resolve a grievance, the logistic regression results did not fully support this hypothesis. Leakage warning behavior, having a co-conspirator, and having a motive involving suicide demonstrated predictive validity in the full model, whereas only leakage warning behavior predicted group membership when the sample was restricted to suspects 19 years old or younger. The variables that were predictive within the model suggest that averted cases may provide more “points of detection” meaning that there were more opportunities for plans to be discovered and reported. The fact that age was not a significant variable in the model reveals that no potential threat to a school should be ignored simply because of the age of the suspect.

### Implications

The response to targeted school attacks demands a public health approach. Prevention, intervention, and recovery policies must be built on evidenced-based research. Accurate and timely data collection, analysis, and reporting is critical to defining the phenomena of targeted school violence, implementing strategies, and evaluating them to determine what works, and how best practices can be replicated.

The ASV database provides a critical source of information regarding school attacks that did not happen. By studying “near misses” we have a unique opportunity to identify risks and protective factors at the individual, school, and community level, and replicate successful prevention and intervention programs. At the individual level, warning behaviors must be identified as early as possible, and interventions put in place to stop violence and support high-risk and high-need students. According to the U.S. Secret Service’s National Threat Assessment Center (NTAC), Behavioral Threat Assessment and Management (BTAM) programs are the best practice for preventing targeted school violence (22). School-based behavioral threat assessment and management teams can identify and address behaviors that may indicate a student is engaging in pathway behaviors and create holistic intervention strategies to prevent violence, address underlying mental health, or other psycho-social challenges, and implement short-and-long-term community-based management plans.

Although prevention efforts directed specifically at potential perpetrators remain paramount, findings from this study suggest that peers, teachers, and other school personnel may have an important role to play in decreasing school shootings. In 2023, the U.S. Department of Homeland Security released a toolkit for strengthening K-12 reporting. The toolkit outlines a 5-part plan to implement within the school setting. The plan includes encouraging bystanders to report wellness and safety concerns, making reporting accessible and safe, providing transparency around actions taken following a report being made, making reporting a part of daily school life, and creating an atmosphere where reporting is valued and respected (37). In Michigan, the State Police, Office of School Safety, has engaged in an ambitious effort to provide behavioral threat assessment training for K-12 schools throughout the state (38).

All members of the school community must be encouraged to say something if they witness concerning behavior and have the means to report their concerns in a safe and secure manner. The data are clear, peers are critical to school violence prevention. Currently, only twenty-nine of the fifty states have a state-level reporting program (39) with varying approaches to the dissemination of information about these programs. More work is needed to establish state-level reporting centers in every state and to ensure the effective, timely and accurate dissemination of threat information to schools, law enforcement, mental health and other stakeholders to prevent targeted school attacks. Current anonymous reporting systems include Michigan’s “Okay2Say” (40) and Colorado’s “Safe2Tell” (41). Alathari et al. (22) found that peers specifically were the most likely to discover a shooting plot and thus, should be the focus of prevention efforts. Few programs exist that address this educational need and ongoing work at UCF RESTORES is examining different methods by which to provide this education.

This study corroborates previous research findings, that also found a high prevalence of depression and suicidal ideation within those who plan and carry out school shootings (7, 9, 10, 35, 36). Therefore, necessary efforts must be made to increase mental health services to youth, as well as, increasing the acceptability of reporting warning behaviors in those struggling with their mental health. Schools must create positive and supportive environments that values each and every member of the school community, prevents bullying and the disparate treatment of students, teachers, and staff. Schools must receive funding and staffing to meet increasing mental health and medical challenges. Additionally, community members must support and know how to connect persons with mental health challenges to the 988 Suicide and Crisis Lifeline and local crisis centers.

Future research should investigate barriers to reporting leakage behavior to proper authorities, including factors influencing the decision to report, and knowledge about where to report the information. Researchers should collaborate with law enforcement to explore the best practices for reporting leakage warning behavior to law enforcement agencies. Lastly, with growing access to guns, safety remains a significant primary prevention effort. Additional training for parents around eliminating their children’s access to firearms is critical [see Langman (8)]. Community stakeholders must work to advance knowledge regarding firearms safety and storage. Mental health practitioners, law enforcement, medical professionals, faith leaders, and other community stakeholders must collaborate with school officials, parents, and students to develop and implement policies and practices to prevent violence and ensure positive outcomes for students and the communities in which they live. As this article demonstrates – targeted school violence can be prevented.

### Limitations

This research study is the first to compare completed school shootings to averted school shootings, but it is not without limitations. Some entries into the database consisted of first person accounts, but much of the data were obtained from open-source media reports. As a result, planned or completed school shootings involving underage perpetrators had much of the data withheld. Therefore, only information regarding age, affiliation to the targeted school, and basic event descriptions are typically reported. More information is available when perpetrators are tried as adults. Additionally, it is unclear how much of the data are fact-checked before publication when utilizing open-source media information. In addition, a single field expert served as the primary coder of motive data. While the decision rules were developed by mass violence researchers, the utilization of a primary coder could have resulted in some unforeseen biases. However, these limitations were considered before initiating this study, and only robust variables were evaluated. Future research studies should focus on attempting to gain more first-person reports and disclosure of more specific information regarding suspect characteristics.

## Conclusion

In summary, the current study represents the largest comparison of characteristics and behaviors that may differentiate completed and averted school shootings. Our hypothesis that averted school shooting suspects would be younger, have an accomplice, have motives related to grievances, and engage in more leakage behavior than suspects who completed school shootings, was partially supported. Averted shootings were more likely to involve perpetrators working with an accomplice, and individuals who engaged in leakage warning behavior. In contrast, completed shootings were more likely to involve a suicide-related motives. Age was not a statistically significant predictor, although it did differentiate in averted versus completed shootings in a simple comparison.

These results highlight where future prevention and research efforts might be most impactful. Efforts should be made to assess ways to increase reporting of potential school shooting plots so that they may be appropriately investigated. This is consistent with findings from previous investigations of school climate that found that schools that were able to thwart a school shooting plot had previously tried to reduce the negative stigma around students reporting concerning behaviors. Due to the low base rate of school shootings, expanding research efforts to include how to educate people of the warning signs of a potential plot may help increase our detection and intervention efforts resulting in more averted plans.

## Data availability statement

The data analyzed in this study is subject to the following licenses/restrictions: the dataset is the property of Safe and Sound Schools and is available upon request. Requests to access these datasets should be directed to info@safeandsoundschools.org.

## Author contributions

AW: Conceptualization, Formal analysis, Investigation, Methodology, Writing – original draft, Writing – review & editing. KA: Writing – review & editing, Conceptualization, Methodology. CB: Formal analysis, Methodology, Supervision, Writing – review & editing. FS: Data curation, Methodology, Writing – review & editing. DB: Conceptualization, Formal analysis, Supervision, Writing – review & editing.

## Footnotes

^1^safeandsoundschools.org
