# Supplementary material for: An evaluation of completed and averted school shootings
Source: Front Public Health. 2024 Jan 9;11:1305286. doi: 10.3389/fpubh.2023.1305286 (PMC10807037; doi:10.3389/fpubh.2023.1305286)
Supplement: Supplementary file 1 [file Table_1.docx]

**Appendix A**

| **Motive** | **Definition** | **Specifiers** | |
| --- | --- | --- | --- |
| Grievances | Any form of grievance, whether it be towards a member of school personnel, students, or just people in general. Cases in which a person had a specific "hit list" are included here. Additionally, in this category are cases that use phrases "hating everyone at the school" in reference to people not based on race or religious affiliation. | - Do not score as grievance if suicidal ideation is present (these cases will be scored as Suicide). - Do not score as grievance if the grievances are related to delusions or hallucinations, or extremist views. | |
| Suicide | Any case where suicide ideation, death by suicide, or suicide by cop was present, and the suspect also intended  to or carried out the killing/injuring of others. This score will be prioritized over other classifications. |  | |
| Mass Murder | Code for any case in which the suspect's goal was to kill a lot of people, but does not indicate it is out of retaliation, and no suicidal ideation is present. Cases can include those that mention performing acts like  "Columbine," "Sandy Hook," or one of the other more famous school shooting cases. | - Do not score as mass murder if the motive is better explained by extremist ideation (score as Extremist Ideation instead). - Do not score as mass murder if there is indication that the suspect intended to die by suicide. - Do not score as mass murder if there is an indication that this person was attempting to seek fame or notoriety. | |
| Fame | Code for any case where the suspect's goal was to kill people to become infamous/famous. | • | Do not score as fame if the motive is for the suspect to take their own life. |
|  |  | • | Do not score as fame if there is no motive |
| Extremist Ideation | Desire to commit an attack against a specific group of people (e.g., based on race, sexuality, gender, or religion) due to extremist affiliation or ideation (i.g., White Supremacists, ISIS, Nazi, Incels, etc.). | • | Do not score if the extremist ideation is based on Delusional Ideation or Hallucinations (score as delusional ideation or hallucinations instead). |
| Delusional Ideation or Hallucinations | Any case in which delusions or hallucinations are present. Cases included are those in which the perpetrator reports hearing "voices," belief that they are doing the work of a  "higher power," or were a  "higher power." |  |  |
| Other | Any cases that contain enough information but do not fit into one of the above categories. |  |  |
| Unknown | Give this code for cases in which there is not enough information to accurately categorize the case. |  |  |
| Do Not Include | Does not meet the criteria of an averted or completed school shooting and should be excluded from the database. |  |  |
